# A Descriptive Phenomenological Study of the Women's Experiences From the Suspicion of Breast Cancer to the Initiation of Treatment

**DOI:** 10.1111/scs.70023

**Published:** 2025-04-20

**Authors:** Rawdha Touzi, Amal Ben Daly, Awatef Ben Amor, Mohamed Ben Dhiab, Christophe Debout

**Affiliations:** ^1^ University of Sousse Faculty of Medicine of Sousse Sousse Tunisia; ^2^ Department of Forensic Medicine Farhat Hached University Hospital, Research Laboratory Sousse Tunisia; ^3^ Department of Biochemistry Farhat Hached University Hospital, Research Laboratory Sousse Tunisia; ^4^ Nurse anesthesia program, IFITS Neuilly‐sur‐Marne France

**Keywords:** breast cancer, descriptive phenomenology, interview, qualitative study, Tunisia

## Abstract

**Introduction:**

Breast cancer is the most common malignancy among women worldwide. However, scientific research has paid little attention to the lived experiences of these women, from the onset of concerning symptoms to the initiation of curative treatment. This study aimed to explore and understand the experiences of Tunisian women, from the appearance of the first symptoms of suspected breast cancer to the initiation of curative treatment.

**Method:**

This descriptive phenomenological qualitative study, inspired by Husserl's philosophy, is the first of its kind in Tunisia to explore the feelings and experiences of women with breast cancer during the early stages of care. Individual interviews were conducted with 13 women diagnosed with breast cancer who had visited the Farhat Hached University Hospital in Sousse. Participants were selected through purposive sampling. Data collection was carried out in September and October 2022. The Giorgi analysis method was used, allowing for the emergence of three main themes.

**Results:**

The analysis of the interviews revealed three themes: Waiting for diagnostic test results, the announcement of the diagnosis, and waiting for treatments. Together, these themes encapsulated the essence of the phenomenon: a balance between vulnerability to various challenges and resilience in managing the journey from diagnosis to treatment, reflecting a life of both struggle and strength. Participants highlighted the psychological, physical and economic challenges they faced and expressed specific needs related to their condition. These needs were particularly evident during the pre‐diagnosis phase, the moment of diagnosis disclosure and the waiting period before treatment began.

**Conclusion:**

The practical implications derived from the results of this study could help inform medical and nursing practices and guide the development of care delivery strategies to better support women with breast cancer.

## 1 Introduction

Breast cancer is a global burden and deeply impacts the lives of many families [1]. It is the most common and deadliest cancer among women worldwide [2].

The most stressful experiences reported by women facing breast cancer include the suspicion of the diagnosis, the practitioner's announcement of the diagnosis, and the waiting period before the initiation of curative treatment [3]. The quality of these critical stages forms the foundation of the patient's journey and significantly shapes her experience, influencing her care pathway and outlook on life [4].

The diagnostic period is often fraught with anxiety and fear. Women frequently express apprehension about undergoing medical investigations, potential pain, prolonged waiting periods, the doctor's demeanour, and the way the results will be communicated [5]. The consultation during which the diagnosis is disclosed becomes a pivotal moment, often shaking the woman's emotional and psychological equilibrium. This phase signifies the end of diagnostic uncertainty and the beginning of an acknowledgment of the disease, marking a major turning point in the patient's life [6].

Following the diagnosis, women face additional medical investigations and consultations, during which they are required to provide informed consent for critical therapeutic decisions [7]. This pre‐treatment phase is characterised by profound cognitive and emotional distress [8], with many women expressing a desperate desire to remove the tumour that has upended their lives [9].

Health professionals play a critical role during this vulnerable period, and they must provide individualised support to meet the unique needs of each patient [10].

In Tunisia, the situation is particularly concerning. The incidence of breast cancer continues to rise, with 30% of malignant tumours in women attributed to this disease [11, 12]. Alarming trends are observed in the coastal regions. For instance, in Monastir, the crude incidence rate (CIR) of breast cancer was reported at 34.25 per 100,000 inhabitants, with the highest rate among women aged 40–59 years (101.7 per 100,000). The age‐standardised incidence rate (ASR) was 39.12 per 100,000, and trends have shown a significant annual increase of 8.4% between 2002 and 2013. Projections suggest a continued rise, with an ASR expected to reach 108.77 per 100,000 by 2030 [13]. Additionally, a significant proportion of breast cancer cases are diagnosed at advanced stages, with a median delay of 6.8 months before consultation [14, 15]. Furthermore, the communication of the diagnosis often occurs without a structured psychological support protocol. Physicians typically disclose the diagnosis alone, without the involvement of social workers or nurses.

Although some research has explored specific stages of the breast cancer journey—such as the pre‐diagnostic period [15, 16, 17], the post‐diagnostic period [18, 19, 20], and the interval between diagnosis and treatment initiation [21, 22]—a comprehensive examination of the overall experience encompassing all these phases remains limited. These periods are interdependent, and the woman's overall experience and emotions are often shaped by her journey from the initial disruption of her healthy life to the acknowledgment of the disease [9].

A deeper understanding of the lived experiences of women with breast cancer during this critical time could provide valuable insights into the challenges they face, as well as their needs and expectations. Such knowledge would be instrumental in guiding effective and patient‐centred interventions.

## 2 Objective

This study aimed to explore and understand the experiences of Tunisian women, from the appearance of the first symptoms of suspected breast cancer to the initiation of curative treatment.

## 3 Methods

A descriptive phenomenological approach was employed for this research. The transversal design was selected to minimise the need for multiple participant engagements, thereby enhancing convenience and feasibility.

Husserl's transcendental phenomenology constitutes the methodological basis of this study and, more specifically, Giorgi's method [23].

### 3.1 Participants and Setting

This qualitative study focused on women in remission from breast cancer who had received treatment at the outpatient consultations of the obstetrics department at ‘Farhat Hached’ University Hospital in Sousse, Tunisia. Participants were selected using purposive sampling to ensure a diverse representation of experiences. Data were collected through in‐depth face‐to‐face interviews during September–October 2022.

Eligible participants included women over 18 years who were in the extended or permanent survival phases after breast cancer treatment, following Mullan's framework [24]. The sample aimed to capture a broad spectrum of experiences by including women with diverse sociodemographic characteristics, such as age, marital status, educational level and occupation. Inclusion criteria required participants to be diagnosed with breast cancer, have completed treatment, and be in remission. Women diagnosed with breast cancer during pregnancy or lactation, those with a history of other cancer types, or those with cognitive, neurological, or psychiatric conditions that could impede interview participation were excluded.

### 3.2 Data Collection

Thirteen participants diagnosed with breast cancer were selected through purposive sampling.

Individual unstructured interviews were conducted in person at locations chosen by participants, including their homes, a café, the hospital garden, or the port of Sousse.

Each participant was interviewed once. Before each interview, participants were briefed about the study objectives, ethical principles and their rights, including voluntary participation and withdrawal. Written informed consent was obtained from all participants.

The interviews, conducted in the Tunisian dialect, lasted between 25 and 62 min. Each session began with a sociodemographic questionnaire to gather information on age, place of residence, marital status, educational level and occupation. The induction question was: ‘Can you tell me what you have experienced since the suspicion of breast cancer until the beginning of treatment?’.

To ensure comprehensive documentation, the interviewer recorded field notes to capture contextual details, participant reactions and reflections. Data collection continued until thematic saturation was reached, determined by the absence of new codes emerging in the final interviews, indicating that sufficient data had been gathered to represent the phenomenon. This aligns with the principle of data saturation commonly used in qualitative research [25].

The interviews were transcribed verbatim in the Tunisian dialect to preserve the participants' exact words and meanings. Then the transcriptions were carefully translated into English, with close attention to preserving the meaning and nuances of the participants' words. The translated transcripts were reviewed multiple times to ensure accuracy and consistency between the original Tunisian dialect and the English translation.

### 3.3 Data Analysis

Giorgi's five‐step content analysis method [23] was used to analyse the research data manually. The analysis process involved:

- Making sense of the whole: The interview transcripts were read multiple times to gain a general understanding of the participants' experiences.
- Determining meaning units: Significant phrases and key expressions were identified and segmented into meaning units. Focus was maintained on preserving the participants' intentions and avoiding imposing external interpretations.
- Transforming meaning units: The identified meaning units were analysed and transformed into psychological expressions that reflect participants' lived experiences.
- Synthesising transformed meaning units: The transformed units were organised and clustered into themes and subthemes based on similarities.
- Final synthesis: A comprehensive description of the phenomenon under study was developed, integrating all identified themes and subthemes. The software Xmind assisted with organisation and visualisation during the analysis. The synthesis provided a faithful representation of the participants' experiences and highlighted key findings relevant to the research objectives.

### 3.4 Rigour and Trustworthiness

To ensure the trustworthiness of the findings, several strategies were applied based on Lincoln and Guba's framework [26]:

- Credibility: Prolonged engagement with participants and iterative review of interview data enhanced understanding. Continuous observation in the field allowed for a deeper understanding of the events and the diverse perspectives of participants. Findings were validated through discussions and comparisons with existing literature.
- Transferability: A diverse sample and detailed contextual descriptions support the applicability of findings to other settings. Thorough descriptions of the study context and participant demographics allow for informed judgements regarding the applicability of results.
- Confirmability: Participant quotes were used to substantiate themes, and a reflective logbook was maintained. Study recordings were securely stored for future verification.
- Dependability: The integrity of data was ensured through systematic documentation of analysis processes. Rigor in data analysis and careful avoidance of premature conclusions contributed to the dependability of the study.

### 3.5 Ethical Considerations

The research was conducted according to the principles of the Declaration of Helsinki [27], ensuring that all participants provided written informed consent. To protect confidentiality, the collected data was anonymized. The study received approval from the Ethics Committee of the Faculty of Medicine in Sousse, under reference number CEFMS 16/2019. Additionally, all audio recordings and transcripts were securely stored to maintain participant privacy.

## 4 Results

### 4.1 Demographic Characteristics

Thirteen women participated in the study, with ages ranging from 31 to 64 years, with a mean age of 46.69 years. The geographical distribution of participants included seven women from Sousse, one from Mahdia, two from Kairouan, one from Sidi Bouzid, one from Monastir and one from Kasserine. Ten of the participants were married with children, two were single, and one was divorced. In terms of education, four had primary education, three had secondary education, and five had university education. One participant had never attended school. Regarding employment, four participants were employed, three were retired, one was a student, and the remaining participants were housewives.

The analysis of the participants' interviews revealed three themes and fifteen subthemes (Table 1).

**TABLE 1:** Themes and subthemes.

Themes	Subthemes
Awaiting diagnostic test results	Delays in starting treatment Effects of family history of cancer Sources of information Emotional responses Reactions of the family environment Expected solutions to save time Patients' needs and expectations
Announcements of the diagnosis	Communication of the diagnosis Emotional impact Coping mechanisms Patients' needs and expectations
Waiting for treatment	Seeking care at public hospitals Patient's feelings and reactions Reaction from the patient's entourage Patients' needs and expectations

In synthesising these themes, the essence of these women's experiences can be understood as a delicate balance between vulnerability to systemic, emotional, and social challenges and their adaptability and resilience in navigating the journey from diagnosis to treatment. It reflects a lived experience characterised by both struggle and strength.

### 4.2 Theme 1: Awaiting Diagnostic Test Results

#### 4.2.1 Delays in Starting Treatment

Delays stemmed from healthcare‐related causes, patient‐related causes and external factors. Healthcare system delays included long waiting times for appointments in public hospitals and diagnostic delays, with some doctors misinterpreting breast symptoms and attributing them to benign conditions. One participant noted: ‘It's been a month since my shoulder hurt, and when I consulted doctors, I was told I had nothing’ (P3). From the patients' perspective, delays also stemmed from a lack of encouragement from their surroundings and failure to notice symptoms: ‘When I raise my hand, it hurts; I had pain in my armpits… I didn't pay attention’ (P2). Financial constraints also played a significant role; for instance, a student participant mentioned being unable to afford medical visits, which led to a delay of several months. Additionally, some women were preoccupied with other competing life events: ‘We were busy with preparations for my daughter's wedding. I knew I had cancer, but… I wanted to finish the wedding’ (P7).

#### 4.2.2 Effects of Family History of Cancer

Participants with a family history of cancer reported being somewhat mentally prepared for the diagnosis. One participant shared: ‘I was already morally prepared because my mother was sick. It wasn't a shock for me’ (P2). Another participant explained that her sister's illness led to the habit of self‐palpating and seeking tests.

#### 4.2.3 Sources of Information

Four women indicated that their suspicion of cancer was influenced by information gathered from the media, online research, and advice from health personnel or family members with similar experiences.

#### 4.2.4 Emotional Responses

The discovery of a breast lump led to feelings of ‘doubt’, ‘stress’ and ‘fear’. Patients reported feeling anxious and impatient while waiting for results, describing the process as a ‘nightmare’. Participants expressed their anxiety as a pervasive emotional state that shaped their perception of time and care.

#### 4.2.5 Reactions of the Family Environment

The reactions of participants' families varied. Some women received strong emotional support, such as encouragement from their family members to continue the medical process. However, one participant's husband, guided by the doctor's advice, hid the diagnosis from her, which caused her emotional distress: ‘My husband didn't tell me, but he came out crying’ (P3).

#### 4.2.6 Expected Solutions to Save Time

A relative working in the healthcare system was a significant asset for some participants, helping expedite procedures. Additionally, some opted for private healthcare despite financial constraints, as one of the patients, after consulting two doctors, opted for the one who offered to perform the surgery at a lower cost.

Another participant highlighted a disadvantage of private healthcare stating: ‘I lost confidence in my gynecologist because I found her unprofessional. She took advantage of my situation, so I switched to another doctor’ (P5).

#### 4.2.7 Patients' Needs and Expectations

Participants consistently expressed a strong need for measures to save time during their diagnostic journey. One participant (P7) suggested the establishment of a dedicated administrative unit specifically for cancer patients to streamline procedures. Another participant proposed implementing a system for assigning numbers during appointments to reduce waiting times, which would enable them to ‘attend to other tasks and errands instead of waiting for hours in the waiting room’ (P13).

The lack of information provided by physicians about their condition emerged as a significant concern. A participant shared ‘I had no information about the disease or chemotherapy’ (P11), underscoring a clear gap in communication during the diagnostic phase.

Additionally, participants highlighted the urgent need for better psychological support. One participant vividly described her emotional turmoil: ‘I was worried, confused, and scared’ (P5), reflecting profound anxiety while awaiting results. She further elaborated: ‘During this whole period, I was on edge; I would be lying if I told you I was comfortable—not at all. I was worried, confused, and scared’ (P5).

### 4.3 Theme 2: Announcement of the Diagnosis

#### 4.3.1 Communication of the Diagnosis

How the diagnosis was communicated to patients varied significantly. One participant criticised the lack of sensitivity, stating: ‘I did not accept the way he told me about the disease… he could have prepared me psychologically’ (P10). Only one patient (P11) reported having access to a psychologist for support.

Many participants felt the information provided was either unclear or insufficient. One woman described a distressing experience where the diagnosis was delivered abruptly in a hallway, leaving little time to process the news: ‘I ran behind him in the hallway and begged him to read my file to see what I had. He told me I had surgery and chemo sessions’ (P9). Another participant recounted an impersonal encounter: ‘The secretary called me. I went to meet her, and I found a doctor with her. They told me I had cancer’ (P6).

In some cases, the diagnosis was communicated to a family member instead of the patient. For instance, one participant shared, ‘My husband was the one who got the result and consulted with the doctor […] then he informed me that the doctor advised surgery to remove the breast’ (P10). These accounts highlight significant variability and, at times, a lack of consideration in how such life‐altering news was conveyed.

#### 4.3.2 Emotional Impact

The timing of the diagnosis evoked profound emotional responses among patients, with terms like ‘shock’, ‘upset’ and ‘collapsed’ frequently mentioned. One participant (P11) described the experience as akin to throwing herself ‘into the void’ while others reported feeling ‘psychologically fatigued’ upon learning of their condition. One patient shared how a technician initially trivialised the mammogram results, providing false reassurance: ‘You can't imagine the joy I experienced. I left her lab and went to see my doctor again with incredible joy’ (P10). However, when the diagnosis of breast cancer was confirmed, she described her reaction as one of ‘hysteria’. Sleep disturbances were common, with several patients experiencing insomnia for consecutive nights.

Unexpectedly, one participant shared that when she heard the diagnosis, she started laughing: ‘When I found out about my illness, I was laughing as if I had been told I had won something, I swear to you, psychologically, I was fine’ (P6). According to her, this reaction seems to be linked to her faith and her personal history with her sisters' illness. She felt she was ‘prepared to have the disease’.

The diagnosis not only triggered personal distress but also raised existential concerns, particularly among mothers. Many worried about their children's future and made arrangements with their mothers or sisters to care for them in case of death. Yet, for one participant, her role as a mother became a source of strength: ‘I had to fight for my children. It was necessary to overcome the disease for them’ (P11).

Relationships were also affected. One participant described her emotional withdrawal from her husband: ‘When he came to sleep next to me, I would not let him’ (P3).

The stigma surrounding the illness led many patients to conceal their diagnosis. For some, the motive was to shield family members from worry, while others sought to avoid societal judgement. One unmarried patient expressed concern about how the diagnosis might diminish her prospects of marriage: ‘The fact that a girl has cancer would decrease her chances of getting married’ (P13). Another participant shared her decision to isolate herself, explaining, ‘When they discover that a person is sick, they do not support her and see her as a dying woman’ (P10).

#### 4.3.3 Coping Mechanisms

Religious faith emerged as a key coping mechanism for most of the participants. One participant explained: ‘I have faith and I always try to have it…I tell myself that life is in the hands of God, …I do not bind sickness to death’ (P11), showing how faith provided her strength during a difficult time.

#### 4.3.4 Patients' Needs and Expectations

Various needs and expectations were highlighted by some participants, starting with a pronounced demand for psychological support. As one participant put it: ‘I needed moral support. I needed everyone to cheer me up … patient psychology is very important’ (P5). Alongside this, the need for clear and comprehensive information became evident. Some participants expressed dissatisfaction with the level of detail provided, particularly concerning their diagnosis and the treatments they were expected to undergo.

### 4.4 Theme 3: Waiting for Treatment

#### 4.4.1 Seeking Care at Public Hospitals

All participants sought care within public hospital settings to initiate curative treatment. They recognised certain advantages, such as affordability, competent nursing staff and reliable medical care. However, many reported significant drawbacks, including perceived neglect, disparities in how medical staff treated patients, and long waiting times for treatment. These challenges were often described as sources of anxiety. One participant reflected: ‘The wait felt endless, and every passing day heightened my fears’ (P9).

#### 4.4.2 Patients' Feelings and Reactions

Participants commonly referenced ‘stress’ and ‘fear’, particularly the fear of losing their breasts. Some participants also described demonstrating resilience during this period.

One patient shared her source of strength: ‘The strength I had was given to me by God. Prayer gave me strength, and I was strong’ (P4).

Decisions related to surgery revealed a spectrum of responses, ranging from refusal to acceptance. For instance, one participant chose total mastectomy despite her doctor's disagreement, explaining: ‘My mother didn't remove her breast when she had breast cancer, and the disease came back. I couldn't take that risk’ (P2).

A few participants turned to the internet to better understand their condition and explore available treatments. For one patient, this research led her to explore traditional medicine and adopt a special diet.

#### 4.4.3 Reactions From the Patients' Entourage

The reactions of family members and loved ones varied widely, shaping the patients' experiences during this phase. For some, family support was a vital source of comfort. As one participant noted: ‘I was always surrounded by my family, and I did not experience my illness alone’ (P6).

Others, however, worried about the emotional toll their illness placed on their children: ‘My children were tired because of me, emotionally and mentally. Their school performance suffered’ (P8). The burden of caregiving often fell on close family members, such as husbands or daughters. Yet, not all experiences were supportive. Two participants reported feeling abandoned. One woman recounted: ‘When my husband found out about my illness, he was terrified of catching cancer. He thought it was contagious, so he left me’ (P8).

#### 4.4.4 Patients' Needs and Expectations

Most participants highlighted several unmet needs and expectations during their wait for treatment. Time was a recurring concern, with many expressing frustrations over delays. One patient emphasised: ‘When doctors diagnose cancer, they should help the patient save time and start treatment before it's too late’ (P12).

The need for psychological support was also evident. Patients wanted empathy and reassurance from their medical team during this vulnerable period. As one participant explained: ‘When a patient is told she has cancer, she needs doctors to stand by her and support her. That's the moment when help is most needed’ (P12).

Some patients reported discrepancies between the information they initially received and the reality of their treatment. One woman shared: ‘I wasn't given clear information. They said we might remove the breast or not because they didn't know what was there. They could have just told me outright what would happen. I also wasn't informed that chemotherapy or radiation might be needed’ (P6). Another participant reflected on her dashed hopes: ‘I was told the nodule could be removed without chemotherapy. I was so happy because I had hidden the truth from my family. But after surgery, it turned out to be malignant, and I had to undergo chemotherapy’ (P13).

Additionally, patients expressed the desire for treatment facilities closer to home to reduce the financial and emotional strain of travelling between cities. Many also wanted access to associations or support groups that could provide guidance, help them navigate their condition and offer a sense of solidarity.

## 5 Discussion

This study provides an in‐depth understanding of the lived experiences of Tunisian women from the suspicion of breast cancer to the initiation of treatment. The findings reveal the profound psychological, physical and economic challenges these women face and the resilience they demonstrate in navigating their journeys.

The findings highlight significant delays between the initial suspicion of breast cancer and the start of treatment, consistent with other studies from Tunisia and similar contexts. Previous research in Tunisia stated that the delay between the discovery of symptoms and the initiation of treatment ranges from 10 days to 2 years [1]. In contrast, the median delay in high‐income countries is between 30 and 48 days [28]. This significant disparity can be partly attributed to failures within the healthcare system, where practitioners may attribute non‐specific symptoms to benign pathologies without adequately exploring the possibility of breast cancer. Diagnostic errors, such as misinterpretation of mammograms or breast biopsies, further exacerbate these delays. Such shortcomings waste valuable time and deprive patients of the opportunity for timely treatment, leaving them feeling neglected and not taken seriously. These delays reflect broader structural inequities and point to critical gaps in healthcare accessibility and efficiency.

These delays stemmed also from patient‐related factors, such as financial barriers, lack of awareness and competing life priorities. Some women may attribute early symptoms to other, less serious conditions or assume that these signs will resolve spontaneously, leading them to delay seeking medical attention [17, 28]. This lack of knowledge often leaves patients feeling uncertain about their situation. These findings align with the study by Doré et al. [16] which reported that many women expressed a significant unmet need for information and stated their lack of understanding regarding the pre‐diagnosis process, including the steps involved in evaluating their condition.

To address these challenges, a dual approach is necessary. This involves strengthening the healthcare system through continuing education for practitioners to enhance diagnostic accuracy [29] and facilitate timely referrals. Additionally, implementing public health initiatives is crucial to promote awareness of breast cancer symptoms and emphasise the importance of early detection [18], particularly in underserved areas. This can be achieved through comprehensive audiovisual media campaigns and the deployment of mobile breast cancer screening units to reach women in underserved and remote areas. These efforts would help reduce diagnostic delays and improve patient outcomes.

Sometimes, healthcare providers opted to withhold information about the diagnosis, believing this approach would protect the patient from distress. Unfortunately, this secrecy often intensified the women's anxiety, leaving them in a state of uncertainty and consumed by doubt.

In this context, article 36 of the Tunisian Code of Medical Deontology [30] states that a serious or fatal prognosis may be withheld from the patient. It should be revealed with caution, though it can generally be disclosed to close family members. However, it is important to recognise that while withholding a fatal prognosis may be appropriate in some cases, this approach does not apply universally, especially in the case of early‐stage breast cancer, which can often be cured. Withholding the diagnosis in such instances can lead to confusion, delays in understanding, and hinder the patient's ability to make informed decisions about their treatment and healthcare journey.

Support from loved ones played a crucial role for some participants, providing emotional comfort and assistance in decision making. Interventions should include educational programmes for family members to help them understand the patient's needs and provide appropriate support.

The importance of time‐saving strategies emerged as a recurrent theme among participants, many of whom initially sought care in the private healthcare sector. Although this choice often expedited access to diagnostic and treatment services, it also imposed a significant financial burden, adding yet another layer of stress and anxiety. Participants highlighted the need for a dedicated administrative unit specifically for patients with malignant tumours. Such a unit could address logistical issues such as appointment scheduling, consultation hours, and reduce delays for tests and examinations, thereby easing the administrative burden on patients.

When the diagnosis was communicated, the participant's suffering was amplified by several factors. The doctor's cold and impersonal attitude, the use of abstract or complex information that exceeded the patients' level of understanding, and the inadequacy of the setting where the diagnosis was announced were frequently reported. Only one participant expressed satisfaction with how she was informed, which may have been due to her sense of comfort, as a relative of hers worked in the same healthcare facility and was familiar with the staff. This exception highlights a broader issue: the diagnosis announcement was almost unanimously described by participants as abrupt and lacking empathy. This lack of sensitivity can partially be attributed to the heavy workload of healthcare providers, which limits the time they can dedicate to each consultation [31]. However, the primary issue lies in the lack of training for practitioners in delivering such critical news [32]. Communicating a cancer diagnosis is inherently challenging for both patients and healthcare providers. To improve this process, diagnosis announcements must be conducted by qualified and well‐trained professionals. These professionals should be supported by a nurse capable of providing empathetic communication, creating a more supportive environment for the patient. Hospital structures must also prioritise the provision of continuous psychological support, beginning at the time of diagnosis and extending throughout the treatment process. This support should be reintroduced during pivotal moments, such as treatment relapses, to address the evolving emotional and psychological needs of patients. By implementing these measures, healthcare systems can significantly enhance the quality of care and emotional support provided to individuals navigating a cancer diagnosis.

Being diagnosed with breast cancer is a life‐altering experience for most women [33]. As seen in other studies [18, 19], the participants described a wide range of emotions upon receiving their diagnosis. Initial reactions ranged from crying to, surprisingly, bursts of laughter. Others reported somatic responses, such as fainting while processing the shock.

Regardless of these varied responses, healthcare professionals must closely monitor patients following the diagnosis to assess their emotional and psychological needs. Providing guidance and support can help patients better assimilate and accept the diagnosis [32]. Among younger participants, some noted avoiding physical contact with their spouses after receiving the diagnosis. Studies have shown that younger women are at a higher risk of experiencing sexual and psychological distress in such situations [34]. This phenomenon warrants further scientific investigation to better understand and address its implications.

In some cases, participants chose to withhold their diagnosis from family members, either to spare their loved one's emotional pain [32] or to avoid being stigmatised or marginalised [16]. Unfortunately, this decision often left these women isolated, overwhelmed by negative emotions, and without the support they needed. Healthcare teams should emphasise the importance of family involvement and encourage patients to seek support from their loved ones, explaining how it can positively impact their journey [32].

If the patient prefers not to involve her family, it becomes even more crucial to provide alternative sources of reassurance and emotional support. Creating opportunities for patients to connect and share their experiences is essential in such cases. Establishing roundtable discussions involving women with breast cancer would offer a valuable platform for exchanging experiences, fostering mutual support and generating insights to improve caregiving practices. This could significantly contribute to empowering patients and refining care strategies.

For many participants, faith and religious practices were a source of solace. Praying and seeking spiritual guidance helped them release emotional pressure and manage their complex feelings. The literature corroborates this finding, noting that religious practices are among the most common coping mechanisms for cancer patients [18, 35]. Given the demonstrated positive effects of spirituality on psychological well‐being, healthcare professionals should consider incorporating these practices into their care strategies to foster optimism and resilience during the diagnosis process [18].

During the treatment waiting phase, participants frequently expressed dissatisfaction with the lack of attention, support and in some cases, mistreatment by healthcare professionals. These findings align with those of Doré et al., where participants similarly lamented the absence of psychological support and the unavailability of professionals to whom they could turn for emotional refuge [16].

In response to dissatisfaction with the information provided during all phases of their care, some participants took on the additional task of seeking information online to better understand their illness. Although online information is readily accessible with a single click, its reliability and quality are not guaranteed. In some cases, it may even be harmful [36]. This underscores the critical need for healthcare providers to deliver comprehensive and accurate information to patients throughout every phase of their care. Another possibility is the creation of accessible communication platforms, such as a telephone helpline, managed by experienced healthcare professionals that would allow patients to ask questions, receive accurate information and be directed to appropriate resources.

Family support remains a vital pillar for patients, playing a fundamental role in helping them navigate the disease's physical, psychological, social and economic impacts and adhere to the required treatments [18]. Most of the participants reported being supported and accompanied by their spouses, who shouldered significant physical, social, economic and emotional burdens of cancer care [37]. However, a few women highlighted the absence of their husbands during this challenging time. For these patients, the lack of spousal support further intensified their distress.

Notably, discussions about family support were more prevalent during the waiting period for treatment. Most patients did not mention such support while awaiting diagnosis or its official announcement. This could be explained by the fact that many had initially concealed their illness and only disclosed it at later stages. This finding reinforces the importance of psychological support provided by healthcare professionals from the earliest stages of disease suspicion.

Although loved ones serve as invaluable sources of support, they are also deeply affected by the disease and often experience psychological repercussions. Caregivers may develop depressive disorders, anxiety and significant stress reactions [38]. These challenges are especially pronounced for caregivers actively involved in patient care, whose psychological distress tends to exceed that of the general population [37]. Therefore, targeted strategies are essential to mitigate the psychosocial, occupational and economic burdens faced by caregivers [39].

## 6 Study Limitations

This study has certain limitations that should be acknowledged, although they do not diminish its overall value. First, the use of a phenomenological approach combined with purposive sampling may influence the scope of the findings. Although this methodology allows for a deep exploration of participants' lived experiences, the results are closely tied to the specific context of the study and may not be directly generalizable to other populations or settings. Additionally, the process of translating the interviews, which were conducted in the Tunisian dialect and later translated into English, may have introduced some challenges. Despite rigorous efforts to preserve the richness and accuracy of participants' accounts, there is a possibility that certain linguistic or cultural nuances were partially lost in translation, which could have subtly influenced the interpretation of the data. Finally, the need to redirect conversations during interviews may have had an impact on the exploration of certain themes. Participants often focused on their experiences during the treatment phase, which occasionally required steering discussions back to the pre‐diagnosis and diagnostic phases to align with the study's objectives. Although these adjustments were necessary to ensure the relevance of the data collected, they may have slightly constrained the natural flow of participants' narratives and limited insights into some aspects of their experiences.

## 7 Conclusion

Through this study, the experiences of 13 women were analysed, shedding light on the psychological, physical and economic challenges they encountered from the onset of breast cancer symptoms to the initiation of therapeutic management. These women expressed distinct needs that became particularly acute during transitional periods, such as the pre‐diagnosis phase and the waiting period before treatment.

The implications of this study extend to several key areas. For the healthcare setting, efforts should focus on minimising delays in diagnosis and treatment, which were identified as major sources of anxiety. Within the Tunisian health system, strategic initiatives should aim to enhance access to timely and compassionate care, streamline diagnostic pathways and ensure resource allocation aligns with patient needs.

In terms of education, healthcare professionals should receive enhanced training in communication and psychological support to bridge existing gaps and adopt a patient‐centred approach that integrates emotional, informational and practical support. This includes providing continuous professional development opportunities.

Future research is needed to investigate structural barriers to timely care, assess the long‐term psychosocial impacts of breast cancer and identify effective strategies for building a more empathetic and responsive healthcare system.

By addressing these challenges, healthcare providers, educators and policymakers can work towards improving the overall experience and quality of care for women with breast cancer in Tunisia.

## Author Contributions

All authors agree with the content of the manuscript. R.T. collected the data, conducted the interviews, and analyzed the data in collaboration with A.B.A. M.B.D. and C.D. supervised and reviewed the study. R.T. and A.B.D. participated in the preparation and drafting of the manuscript. All authors contributed to reading, revising, and approving the final version.

## Conflicts of Interest

The authors declare no conflicts of interest.

## Data Availability

The data that support the findings of this study are available from the corresponding author upon reasonable request.
